# Supplementary material for: Understanding the feasibility of home-based rehabilitation in kidney transplant recipients: A mixed-methods interpretation
Source: PLoS One. 2025 Dec 19;20(12):e0336620. doi: 10.1371/journal.pone.0336620 (PMC12716730; doi:10.1371/journal.pone.0336620)
Supplement: S1 File — (DOCX) [file pone.0336620.s001.docx]

**Supplementary material S1 – final interview topic guide and 3-month follow-up call guide**

**ESCERT topic guide V2**

**Introduction**

“Thank you for agreeing to speak to me. The reason I have asked you to take part in this discussion is that I would like to understand more about your experiences of the exercise programme. This is important because very little is currently known about peoples’ views on this subject.

Before we begin I would like to remind you that whatever you say here will be anonymous. Your names and personal details will not be mentioned in any report. I am using a digital recorder to record our conversation because it is difficult for me to write down everything you say. This will also enable me to give you our full attention and listen to what you have to say.”

Any questions before we start?

**General Views on the study**

1. Firstly, we would like to talk to you very generally about how you found the study. To start us off, can you tell us a little about what you thought about the study?

- *recruitment, any concerns or worries when initially approached – COVID or other*
- *randomisation*
- *study visits/ follow-up*
- *would they like to know results of trial at the end? How would it be best to deliver this information*

*Prompt for positive and negative experiences*

**Views on the outcome measures**

2. Can you tell me what you thought about the assessments you did as part of this study?

- *Cardiac MRI*
- *Blood and urine collection*
- *Max testing on bike*
- *TUAG*
- *STS60*
- *Balance*
- *Gait speed*
- *Lower limb strength*
- *Accelerometer for 7 days*
- *US of quads*
- *BIA*
- *Questionnaires – IPOS, SF12,FACIT-F, Pittsburgh sleep, PAM, Health literacy, DASI, GPAQ (8 total)*

*3.* Which of the measurements felt most relevant/ important to you?

4. Where there any that were less relevant/ important?

5. Where there any that were missing that you thought should be included?

6. What did you think about the number of measurements taken?

**Views on exercise**

7. Can you tell me about your past experiences with exercise?

8. Have you ever taken part in home-based exercise before?

- *If yes, when? What? Why?*
- *How does your experience in this study compare?*

**Views about the exercise programme**

9.Can you tell me what you thought about the introduction to the programme

- *introductory call*
- *Videos*
- *level of understanding of programme after the introduction*
- *Diary*

10. If were to run this study again, how do you think we could improve the introductory session?

*- face to face supervision*

11. Can you tell us how you found the exercise programme (the 12 weeks of home-based training)

- *resistance exercises*
- *aerobic exercises*
- *barriers to taking part*
- *facilitators to taking part*

12. What were you expecting from taking part in this programme?

13. Were your expectations met?

14. What did you think about the support you were offered during the programme?

15. Having taken part in the programme, have your feelings about exercise changed in any way?

- *Prompt positive and negative*

16. If we were to offer the exercise programme to other people with a kidney transplant in the future, can you suggest any ways in which we can improve it?

Quality of life

We are interested in this study to see if exercise has any effect on participants quality of life and overall health

17. What does **quality of life** mean to you?

18. Has this changed

*- over your life time*

*- since your diagnosis?*

19. Has this changed since taking part in this programme?

20 .Has the exercise programme affected your daily living in any way?
*- If so how*

21.How would you describe your current **physical and mental health**?

22. Has it changed in any way since taking part in the study.

- *If so how?*

23. Have there been any other benefits of taking part that you have noticed?

24. Have there any drawbacks or negatives from taking part?

25. Do you think you will continue to exercise now the study has finished?

- *Why/Why not*

26. Who do you feel would be in the best position to support you after the programme?

- *other patients,*
- *friend and family,*
- *physiotherapist,*
- *doctors,*
- *fitness instructors*
- *someone else?*
  What does this support look like?

27. Would you recommend this programme/ study to other transplant recipients?

- *What types of things would you tell them about it?*

29. Is there anything else you would like to say, or did you expect me to ask you something that I haven’t?

**ECSERT 3-month follow-up call questions**

‘Are you currently undertaking any form of weekly exercise?’

‘How often are you undertaking exercise (sessions per week)?’

‘What types of exercise are you currently doing?’

‘If you part of the intervention group, did the exercise programme inform your current exercise habits?’

**Supplementary material S2 – exercise programme engagement**

**S2 Participant engagement with the ECSERT exercise programme**

| Sex | Age (years) | Daily moderate physical activity (min) | Total # aerobic sessions | Total # resistance sessions | % of max aerobic sessions* | % of max resistance sessions* | Total # sessions (wk 1-6) | Total # sessions  (wk 7-12) |
| --- | --- | --- | --- | --- | --- | --- | --- | --- |
| male | 26 | 65.67 | 61 | 24 | 169 | 100 | 45 | 40 |
| female | 49 | 70.17 | 35 | 24 | 97 | 100 | 29 | 30 |
| male | 52^#1^ | 51.81 | 35 | 24 | 97 | 100 | 31 | 28 |
| female | 58 | 16.18 | 20 | 19 | 56 | 79 | 14 | 25 |
| female | 44 | 152.98 | 30 | 13 | 83 | 54 | 23 | 20 |
| female | 43 | 125.95 | 39 | 20 | 108 | 83 | 33 | 26 |
| male | 65 | 64.18 | 53 | 26 | 147 | 108 | 41 | 38 |
| female | 72 | 76.12 | 23 | 24 | 64 | 100 | 22 | 25 |
| female | 55 | 36.40 | 58 | 16 | 161 | 67 | 39 | 35 |
| male | 62 | 22.82 | 35 | 22 | 97 | 92 | 27 | 30 |
| male | 47 | 111.21 | 35 | 19 | 97 | 79 | 29 | 25 |
| male | 52^#2^ | 108.55 | 26 | 24 | 72 | 100 | 26 | 24 |

*max sessions refers to the maximum instructed per week; 3 for aerobic, 2 for resistance. However, participants

could choose to record more.

**Supplementary material S3 – additional quotations for themes and subthemes**

**Table S3.1 Themes relating to the ECSERT trial design and procedures**

| **Theme** | **Example quotations** |
| --- | --- |
| **Perceptions of recruitment and randomisation** | |
| COVID-19 influence | "...if it was at the beginning when Covid first started, when the cases were high, there was no vaccines, I would have been worried. You know I would have been a bit sceptical about coming in, but because obviously I was double vaccinated by then, it had been over a year since I’d had the transplant, I’d already had Covid so I thought you know I felt fairly safe because all the staff had masks on, I had my mask on so yeah, so I didn’t feel frightened or anything or worried or anything. I did feel quite safe." (Female, aged 43) |
| Expectations of randomisation | "I did, yeah, so I really, really wanted to be in the exercise group because I really wanted to get fit. So I didn’t want to wait like twelve weeks, I wanted to use it as an opportunity." (Female, aged 55) |
| **Reasons for taking part** | |
| Desire for physical benefits | "Well just more strength in my arms and my legs and also the heartrate would be better, you know, and the breathing, so yeah, and a little bit more stamina I think." (Female, aged 72) |
| Exploring exercise | "It [exercise] was something that I wanted to do but never found the time to actually – it was just that kickstart really, the plan in place, like ‘ah, this is what you need to do, this is how you do it, these are all the resources available that you could use" (Male, aged 26) |
| Additional health monitoring | "Yeah, it was for myself because of this pain, stopped me getting fit for about three months. I wondered if there was anything wrong with my heart area and that and there’s nothing there. It’s all fine." (Male, aged 62) |
| **Outcome measure acceptability** | |
|  | "I would say the one where – the bike ride [CPET] was the one where I could actually see physical improvements, so that’s the one where I thought OK, well actually this is – because I knew what I felt and how I was afterwards on the initial one and that’s the one where I could see a clear difference in OK, yeah, there has been a change in my stamina etc." (Male, aged 26) [re: CPET] |
|  | "The bike because I do like my bike, I like going to the gym on the exercise bike, I do try and hit targets and know when I've had a good time, so to speak, that was important and I did come home and share my feelings with the family on that." (Male, aged 52^#1^) [re: CPET]  “…being the MRI scan it didn’t bother me at all but it was just the bit when they had, you know when your heart’s beating fast like it’s exercising, that’s the only bit that worried me.” (Female, aged 43) [re: MRI] |
|  | "I was going to say the stand up, sit down one as well because anything that really takes you to the edge of your sort of fitness level seems useful, and both of those two did, because at the end of, even though it was just a minute of stand up, sit down, you’re absolutely [shattered] by the end of it. So yeah, both of those two I think." (Male, aged 47) [re: STS-60] |
|  | "Oh the walking up and down to a chair and back, you walk three and a half paces, turn round, walk three and a half paces back and sit down. That one in three, I couldn’t see that one…just the walking up and down seemed strange." (Male, aged 65) [re: TUAG] |
|  | "Well I sat down at the kitchen table to do them so that I wasn’t just kind of not reading them properly, but I systematically worked my way through and that was fine, but I don't think you could just sit on the sofa and do them, you need to set yourself up properly to do them, or I certainly did, so that you really did think about what you were ticking." (Female, aged 58) [re: questionnaires] |
|  | "Well, some of the questions were so obtuse that it was just give an answer, just give an answer and don’t care." (Male, aged 65) [re: questionnaires] |

**Table S3.2 Acceptability of the exercise aids and programme delivery**

| **Component** | **Example quotations** |
| --- | --- |
| Exercise diary | "Yeah it was pretty straightforward. I like, it wasn’t really hard because I got the booklet on the induction and the exercises didn’t seem, they seemed pretty straightforward to be honest. I could follow it easily through…I think the pictures pretty much, from my aspect I think the pictures on the booklet demonstrated what was needed and there was wording on it so it pretty much made sense." (Male, aged 26) |
| Instructional videos | "I did actually, I used to put a video on whenever I used to do weight lifting I used to keep it on with me so I don't forget anything." (Female, aged 49) |
|  | "Yeah, I think that trunk one where you’re rotating, it took me a while to work out because it was like without twisting your hips or something. There was some elements of it that was confusing written down but the video helped a bit to understand what was going on." (Male, aged 47) |
| RPE scale | "I found it all right. I just treated it as a slightly vague thing because it’s all a bit qualitative is the word you know. It can be hard to judge but it’s like “Well that felt a bit hard. Fifteen”. I didn’t sweat the details too much. I’d look at what I said last week and tried to think “Well was it harder, did it seem harder than that? Yeah, say fifteen” you know. It didn’t cause me any stress but I suspect it’s not the most accurate scale anyway…" (Male, aged 47) |
| Exercise sessions (home-based delivery) | "…probably what would have done me with the weights, not the aerobic side, but say they said ‘right for this study you’ve got to meet once a week, come into, I don't know, the hospital or a hall somewhere, and we’re going to get a lot of transplants together and we’re going to maybe do some little aerobic or concentrate on weights and do that’. I would have gone to that and would have done that quite happily because I would have known I would have had to have been there at a time and do it, so you would have naturally done that. So if you had – or even twice a week if you had to go somewhere and do it – I probably would have done that more." (Female aged 44) |

**Table S3.3 Barriers and facilitators to exercise session completion and maintenance of activity**

| **Theme** | | **Example quotations** |
| --- | --- | --- |
| **Barriers to exercise session completion** | | |
| Finding the motivation, making the personal commitment | "Just trying to stick to it, yeah. I think that’s a personal thing though rather than actually what the exercise is. It’s just one of those things where there’s no one that’s going to push you to do it. It’s that you’ve just got to do it and that’s one of the, that’s my personality in terms of yeah I could be, even now I could be more motivated to do it, yeah." (Male, aged 26) | |
|  | "I used to find it hard motivating myself doing the weights because I was doing it at home. I work all day, I come home at half 4 and I think I’ve got these weights day to day, you know like at the beginning it wasn’t too bad, the first couple of weeks because it was something new and then after that it was like ‘oh these weights’!" (Female, aged 43) | |
| Frequent illness and other health conditions | "The only issue I had was that I do have osteoporosis…I felt that it was pulling my arm and I was really scared because I thought are my bones going to break, am I going to end up like breaking my arms doing this. So I was very – so the thing with the weights was that I was very scared to go higher than like 2 kilos and I would be still now, you know, I don’t think oh let’s try going up, I think well I'm worried in case I break my arm because I've seen it happen with my foot." (Female, aged 55) | |
|  | "I found it quite hard some of them, like I couldn’t like with my right arm, I couldn’t manage like the reps because my right arm, my fistula arm. And I’m left-handed as well so my left hand is dominant and it’s stronger so I could, my left arm I could do 3kg, I could do like 5 reps of 3kg on my left arm where on my right arm I just managed one rep, second rep I’ll just about do it and that’s it. You know my arm’s gone, it’s dead." (Female, aged 43) | |
| **Facilitators to exercise session completion** | | |
| Seeing change and preserving health | "Oh look at me doing sixteen reps instead of ten, oh god, yeah, no, it’s the motivation that I'm preserving what health I have, promoting a better outcome are the reasons why I do exercise, otherwise I would not bother, if I didn’t have to, I wouldn't." (Female, aged 58) | |
| Getting into a routine and the flexibility of home-based exercise | "...like I’ve just done 20 minutes, half an hour in the gym so Tuesday and Thursday was weights and then Bake Off came on and I need to change my days now because I watch Bake Off. So sometimes I used to put the telly on and I’d do my weights while watching." (Female, aged 43) | |
| Variety of options | "I found doing that, my hips were a bit sore afterwards, so I didn’t carry on with that one for example, so I swapped that with another one." (Female, aged 44) | |
|  | "Not giving me a choice, would have just said these are the six or eight ones and you have to do...because I felt like I went for the easier ones..." (Female, aged 43) | |
| The importance of progression | "With 4kg I managed to do five but we did 3 kg I used to do seven...But if I had to go like another two, if I had two more weeks then I would have gone up to six or seven with even 4kg as well." (Female, aged 49) | |
| Obligation to complete and being monitored | "To have a target and be motivated to not just be kind of doing it for myself but somebody was watching me, very tentatively, watching me do it..." (Female, aged 58) | |
| The importance of support | "Actually [clinical trials facilitator] told me that whenever I needed to talk to her she said just send me a message and I will call you, so that was quite helpful..." (Female, aged 49) | |
| **Maintenance of activity** | | |
| Desire to Continue Exercise | “I'm going to repeat it purely because I want to prove to myself that I can be better because I really do want to get more healthy, so kindly [clinical trials facilitator] gave me another exercise book to restart and do that and I will do it but I want to start when I know that I can – to see if I can make a better difference.” (Female, aged 55) | |
| Continuation Methods | “Yeah it has changed the way, yeah it’s just changed the way, like my normal routine. I’ve sort of tried to stick to what I was doing on the 12-week programme so yeah, which has been quite good and I’ve been enjoying it.” (Male, aged 26) | |
| Small but Maintainable Changes | “Since the study I really kept up with the exercise and I am getting so much better. I couldn’t go on the cross trainer because I could only do like 2 mins and my legs would just start aching. Now like at least 15 mins I can be on the cross trainer or even longer. But I do feel like I have become stronger in that way.” (Female, aged 43)  “I’ve not done my weights because I didn’t enjoy that anyway so [laughs]. What I have kept up is when I was doing the walk to school on a few days I’ve been going the longer way around home so I’ve still been doing that.” (Female, aged 44) | |
| Challenges of Continued Exercise | “I had more time to do extra things, you know to go for additional, you know like doing the cardio half an hour, 40 minutes and being able to walk, I had that time whereas now it’s either/or, one or the other. I can’t do both any more, you know with going back to work full time. It’s either go for a walk or do half an hour 40 minutes in the gym, the treadmill and that’s it.” (Female, aged 43) | |
| Barriers of Health and Time | “I’ve had a bit of a setback. I picked up a bug, vomiting and diarrhoea. It just came on all of a sudden…it carried on 2, 3, 4, 5 weeks. Because of that I have lost weight and become really tired.” (Female, aged 43)  “When I am getting busy it is getting hard for me…I’ve started again but in between I used to do the gardening so it’s not that I haven’t done anything I have done it but not you know in the schedule how I used to do it, you know we did training sessions. I wanted to keep it in my routine but sometimes when things happen you can’t carry on can you.” (Female, aged 49) | |

**Supplementary material S4 – factors that participants identified that would help with physical activity adherence or programme continuation**

**Table S4 Factors that participants identified that would help with physical activity adherence or programme continuation**

| **Suggestion** | **Illustrative quotation** |
| --- | --- |
| Repeating the CPET test | “Just doing that cycling trial again! Oh yeah by the way you’ve got to do the cycling trial again in three months’ time. I think that would be really like – oh yeah, you’ve got to improve on what you’ve done before.” (Male, aged 26) |
| Phone calls | “Yeah, if I can get a phone call every now and then to find out how I am doing and all that…” (Female, aged 49) |
| Equipment provided | “Yes some kind of equipment at home because I don't want to go into the gym because I know – if I joined a gym I have – I don't think I can manage with – because I am not working at the moment I don't have any kind of income of my own and I don't want to put a burden on my husband, so I just want to do something at home, so I might get some kind of equipment, you know, I can carry out all the exercise at home.” (Female, aged 49) |
| Yearly review | “I wonder whether a yearly review where we went back and tested a few of the strength things that we've done, like the up and down on the chair exercise, you know, see whether that was maintained after twelve months or that sort of time period…I think it would be better face to face like we did the second lot of review, when we had the second one, because otherwise you kid yourself, don't you, you say ‘well I did 25’, you know, when you’ve really only done 20 or I think it’s human nature, you let yourself off or you add one on rather than take one off, round it up to the nearest five, all this sort of crazy stuff goes on doesn’t it” (Female, aged 58) |
| Additional exercise support | “I think, I don’t know, maybe like you know that the doctors or a health nurse, somebody, I think it would be nice if like somebody would you know like keep track of you, you know like keep you motivated, anyone like my husband normally, he supports me quite a lot.” (Female, aged 43)  “So something a bit like a dietician but for exercise. I suppose that would be a physio but even physios are, they’re massively oversubscribed aren’t they really? They’re really useful humans. But yeah someone connected with the nephrology department in some way but they could even be relatively junior that just sort of knows you know, the sorts of things are sensible to do and can give you the whole kind of exercises.” (Male, aged 47) |
| Dietary support combined with exercise | “…diet and exercise, so the two of them. And maybe whether they’re actually too pushed at the moment to take that on, but it would be beneficial, if people are struggling, to go on a diet programme with that exercise; they might lose a load of weight.” (Male, aged 62) |
| Group sessions | “…but if it is in a little group they can do it together, that will be a brilliant idea…I don’t mind, other people can meetings, we can make more friends, you know, that’s the main things.” (Male, aged 52^#2^) |
