# Supplementary material for: Understanding the feasibility of home-based rehabilitation in kidney transplant recipients: A mixed-methods interpretation
Source: PLoS One. 2025 Dec 19;20(12):e0336620. doi: 10.1371/journal.pone.0336620 (PMC12716730; doi:10.1371/journal.pone.0336620)
Supplement: Attachment — Submitted filename: PONE-D-25-40533 - response to reviewers comments.docx [file pone.0336620.s003.docx]

**Point-by-point answers to reviewer comments**

**Ref.:** PONE-D-25-40533

**Understanding the feasibility of home-based rehabilitation in kidney transplant recipients: a mixed-methods interpretation**

As requested, we provide a **point-by-point answer to the Reviewers' comments** below. We have also submitted a **corrected version** with additions underlined in red and ~~deletions crossed out in blue~~, and a **clean version** of our revision without the corrections still marked in the text.

We thank the reviewers for their comments and feedback to improve our manuscript.

**Reviewer 1**

Clarity on clinical implications – Suggest, in the final discussion, how the findings can be translated into concrete recommendations for home-based rehabilitation programs in different healthcare systems.

**Thank you – we have included a clinical implications section to the discussion which hopefully highlights the key points from the manuscript.**

Qualitative data – Although the justification for not making full transcripts available is valid, the authors could strengthen transparency by highlighting criteria for theoretical saturation and providing additional examples in the supplementary material.

**Thank you. We had added detail in the qualitative data collection and analysis sections to outline the approach we used to ensure as close to saturation as possible was achieved. We feel the excerpts both within text and within the supplementary material provide a solid overview of the data (e.g., 2-3 examples per theme/subtheme).**

Generalizability – More clearly acknowledge that the findings may reflect a group already motivated to exercise, and that applicability to less engaged recipients still needs to be tested.

**Thank you, we have expanded the limitations: ‘***As with most trials of physical activity, it is likely that the sample were somewhat already motivated to exercise and therefore the results may not be generalisable. Effort should be made to engage participants who do not participate in any form of physical activity as the applicability to this subgroup of the population remains uncertain.’*

**Reviewer 2**

Thanks for submitting your valuable manuscript, important topic very good and detailed methodology.

**We would like to thank the reviewer for their kind comments.**

Adding more objective measures is highly recommended please add this to your recommendations, also longer follow up period .

**Thank you, we have refined the table to include these.**

**Additional journal requirements**

1. Please ensure that your manuscript meets PLOS ONE's style requirements, including those for file naming.

**We have made changes in line with the recommendations.**

2. We note that the grant information you provided in the ‘Funding Information’ and ‘Financial Disclosure’ sections do not match.

When you resubmit, please ensure that you provide the correct grant numbers for the awards you received for your study in the ‘Funding Information’ section.

**We have amended the relevant sections.**

3. Thank you for stating the following financial disclosure:

“ Kidney Research UK”

Please state what role the funders took in the study. If the funders had no role, please state: "The funders had no role in study design, data collection and analysis, decision to publish, or preparation of the manuscript."

If this statement is not correct you must amend it as needed.

Please include this amended Role of Funder statement in your cover letter; we will change the online submission form on your behalf.

**Please update the statement to: The funders had no role in study design, data collection and analysis, decision to publish, or preparation of the manuscript.**

4. We noted in your submission details that a portion of your manuscript may have been presented or published elsewhere.

“Feasibility results (previously published) are provided within the manuscript to provide the joint display of quantitative and qualitative (new) results. This prevents the reader having to refer back.”

Please clarify whether this [conference proceeding or publication] was peer-reviewed and formally published. If this work was previously peer-reviewed and published, in the cover letter please provide the reason that this work does not constitute dual publication and should be included in the current manuscript.

**The work referred to is previously peer-reviewed and published however it does not constitute dual publication as these results are not presented in their entirety and are not presented as new work. The previous (key ones only) results are there only as a background to the new analysis. The mixed methods interpretation, qualitative data, and recommendations for future trials have not been published before. If we removed the feasibility data the reader would have to refer back to another publication which does not allow for an easy read or interpretation.**

**I am not sure it fully constitutes previously published work but we wished to be open and honest in our answer.**

**Please see this publication for another example of this way of displaying supportive mixed methods data: https://pubmed.ncbi.nlm.nih.gov/39698363/**

5. We note that you have indicated that there are restrictions to data sharing for this study. For studies involving human research participant data or other sensitive data, we encourage authors to share de-identified or anonymized data. However, when data cannot be publicly shared for ethical reasons, we allow authors to make their data sets available upon request. For information on unacceptable data access restrictions, please see http://journals.plos.org/plosone/s/data-availability#loc-unacceptable-data-access-restrictions.

Before we proceed with your manuscript, please address the following prompts:

a) If there are ethical or legal restrictions on sharing a de-identified data set, please explain them in detail (e.g., data contain potentially identifying or sensitive patient information, data are owned by a third-party organization, etc.) and who has imposed them (e.g., a Research Ethics Committee or Institutional Review Board, etc.). Please also provide contact information for a data access committee, ethics committee, or other institutional body to which data requests may be sent.

b) If there are no restrictions, please upload the minimal anonymized data set necessary to replicate your study findings to a stable, public repository and provide us with the relevant URLs, DOIs, or accession numbers. Please see http://www.bmj.com/content/340/bmj.c181.long for guidelines on how to de-identify and prepare clinical data for publication. For a list of recommended repositories, please see https://journals.plos.org/plosone/s/recommended-repositories. You also have the option of uploading the data as Supporting Information files, but we would recommend depositing data directly to a data repository if possible. Please update your Data Availability statement in the submission form accordingly.

**As the work within the manuscript is qualitative in nature and data is derived from interview transcripts, we cannot make the whole transcripts readily available. Participants consented only to quotations being used within published materials. We have provided as many additional quotations as possible in supplementary materials to support our interpretations.**

6. Please include your full ethics statement in the ‘Methods’ section of your manuscript file. In your statement, please include the full name of the IRB or ethics committee who approved or waived your study, as well as whether or not you obtained informed written or verbal consent. If consent was waived for your study, please include this information in your statement as well.

**Thank you, we have included this within the methods.**

7. We note that there is identifying data in the Supporting Information file <ECSERT Qual Supplementary Material_V1.docx>. Due to the inclusion of these potentially identifying data, we have removed this file from your file inventory. Prior to sharing human research participant data, authors should consult with an ethics committee to ensure data are shared in accordance with participant consent and all applicable local laws.

Data sharing should never compromise participant privacy. It is therefore not appropriate to publicly share personally identifiable data on human research participants. The following are examples of data that should not be shared:

-Name, initials, physical address

-Ages more specific than whole numbers

-Internet protocol (IP) address

-Specific dates (birth dates, death dates, examination dates, etc.)

-Contact information such as phone number or email address

-Location data

-ID numbers that seem specific (long numbers, include initials, titled “Hospital ID”) rather than random (small numbers in numerical order)

Data that are not directly identifying may also be inappropriate to share, as in combination they can become identifying. For example, data collected from a small group of participants, vulnerable populations, or private groups should not be shared if they involve indirect identifiers (such as sex, ethnicity, location, etc.) that may risk the identification of study participants.

Additional guidance on preparing raw data for publication can be found in our Data Policy (https://journals.plos.org/plosone/s/data-availability#loc-human-research-participant-data-and-other-sensitive-data) and in the following article: http://www.bmj.com/content/340/bmj.c181.long.

Please remove or anonymize all personal information (<Age and Participant ID>), ensure that the data shared are in accordance with participant consent, and re-upload a fully anonymized data set. Please note that spreadsheet columns with personal information must be removed and not hidden as all hidden columns will appear in the published file.

**We have removed the individual characteristics table completely as Table 1 provides average values. We have adjusted S2 Table to include only sex, age and adherence to the exercise trial only.**

8. We note that this data set consists of interview transcripts. Can you please confirm that all participants gave consent for interview transcript to be published?

If they DID provide consent for these transcripts to be published, please also confirm that the transcripts do not contain any potentially identifying information (or let us know if the participants consented to having their personal details published and made publicly available). We consider the following details to be identifying information:

- Names, nicknames, and initials

- Age more specific than round numbers

- GPS coordinates, physical addresses, IP addresses, email addresses

- Information in small sample sizes (e.g. 40 students from X class in X year at X university)

- Specific dates (e.g. visit dates, interview dates)

- ID numbers

Or, if the participants DID NOT provide consent for these transcripts to be published:

- Provide a de-identified version of the data or excerpts of interview responses

- Provide information regarding how these transcripts can be accessed by researchers who meet the criteria for access to confidential data, including:

a) the grounds for restriction

b) the name of the ethics committee, Institutional Review Board, or third-party organization that is imposing sharing restrictions on the data

c) a non-author, institutional point of contact that is able to field data access queries, in the interest of maintaining long-term data accessibility.

d) Any relevant data set names, URLs, DOIs, etc. that an independent researcher would need in order to request your minimal data set.

For further information on sharing data that contains sensitive participant information, please see: https://journals.plos.org/plosone/s/data-availability#loc-human-research-participant-data-and-other-sensitive-data

If there are ethical, legal, or third-party restrictions upon your dataset, you must provide all of the following details (https://journals.plos.org/plosone/s/data-availability#loc-acceptable-data-access-restrictions):

1. A complete description of the dataset

2. The nature of the restrictions upon the data (ethical, legal, or owned by a third party) and the reasoning behind them

3. The full name of the body imposing the restrictions upon your dataset (ethics committee, institution, data access committee, etc)

4. If the data are owned by a third party, confirmation of whether the authors received any special privileges in accessing the data that other researchers would not have

5. Direct, non-author contact information (preferably email) for the body imposing the restrictions upon the data, to which data access requests can be sent

**Participants consented to excerpts of interview responses being published. There are no full transcripts within the manuscript or supplementary materials. There is a topic guide of questions but with no responses. We have checked quotations/excerpts do not contains identifiable data. No full transcripts can be shared.**

9. Please include captions for your Supporting Information files at the end of your manuscript, and update any in-text citations to match accordingly. Please see our Supporting Information guidelines for more information: http://journals.plos.org/plosone/s/supporting-information.

**We have updated the manuscript with these.**

10. If the reviewer comments include a recommendation to cite specific previously published works, please review and evaluate these publications to determine whether they are relevant and should be cited. There is no requirement to cite these works unless the editor has indicated otherwise.

**There were no recommendations provided.**

11. Please review your reference list to ensure that it is complete and correct. If you have cited papers that have been retracted, please include the rationale for doing so in the manuscript text, or remove these references and replace them with relevant current references. Any changes to the reference list should be mentioned in the rebuttal letter that accompanies your revised manuscript. If you need to cite a retracted article, indicate the article’s retracted status in the References list and also include a citation and full reference for the retraction notice.

**We have made no changes to the reference list.**
